# Potential of the Job Demands and Accommodation Planning Tool for Individuals Working With Mild Cognitive Impairment or Dementia

**DOI:** 10.1177/14713012251374204

**Published:** 2025-08-28

**Authors:** Katherine Bak, Kristina Kokorelias, Jennifer Boger, Louise Nygård, Mervi Issakainen, Anna Mäki-Petäjä-Leinonon, Ann-Charlotte Nedlund, Charlotta Ryd, Arlene Astell

**Affiliations:** 1 7938 Department of Psychology, University of Toronto, Toronto, ON, Canada; 2 KITE Research Institute, University Health Network, Toronto, ON, Canada; 3 Department of Occupational Sciences & Occupational Therapy, University of Toronto, Toronto, ON, Canada; 4 Geriatrics, Sinai Health, Toronto, ON, Canada; 5 8430 Systems Design Engineering, University of Waterloo, Waterloo, ON, Canada; 6 Technology for Independent Living, Research Institute for Aging, Waterloo, ON, Canada; 7 27106 Department of Occupational Therapy, Karolinska Institutet, Stockholm, Sweden; 8 163043 Law School, University of Eastern Finland, Joensuu, Finland; 9 4566 Department of Health, Medicine & Caring Sciences, Linköping University, Linköping, Sweden; 10 225341 Research Department, Stockholm Gerontology Research Center, Sweden; 11 School of Psychology, Northumbria University, Newcastle-upon-Tyne, UK

**Keywords:** workforce, cognitive decline, job demands, workplace adjustments, young onset dementia, mild cognitive impairment

## Abstract

Individuals living with young onset dementia or mild cognitive impairment are typically working when cognitive decline emerges. Inability to maintain job demands can lead to loss of employment, loss of purpose and identity, financial instability, and withdrawal from social networks. The Job Demands and Accommodation Planning Tool was developed to identify workplace supports for individuals with episodic disabilities. The aim of this study was to explore the applicability of the Job Demands and Accommodation Planning Tool for individuals working with young onset dementia or mild cognitive impairment. Semi-structured interviews were conducted with 23 individuals: 11 in Canada, 7 in Finland and 5 in Sweden, where they described job demands and workplace challenges they faced. A secondary analysis of interview data was conducted using content analysis and a framework approach to code, categorize, and examine overlap with the Job Demands and Accommodations Planning Tool. Results showed that the Job Demands and Accommodation Planning Tool aligns well with the experiences of individuals living with young onset dementia or mild cognitive impairment. The Job Demands and Accommodation Planning Tool could inform workplace supports for people with young onset dementia or mild cognitive impairment to maintain their presence in the workforce.

## Introduction

Difficulties completing work tasks is often an early indicator of emerging cognitive impairment. Young onset dementia refers to individuals diagnosed with dementia before the age of 65, whist mild cognitive impairment is a milder form of cognitive alteration, which may increase dementia risk (Alzheimer’s Association, 2022). Although young onset dementia and mild cognitive impairment are distinct conditions, both can significantly impact an individual’s ability to work, and both require workplace accommodations to maintain employment.

Challenges completing work tasks vary according to the cognitive changes experienced by the individual, but are often first noticed in situations that require learning new material, or remembering routines, appointments or meetings (Chaplin & Davidson, 2016; Keady & Nolan, 1994; McCulloch et al., 2016; Ohman et al., 2001; Ritchie et al., 2015; Ritchie et al., 2018; Wahab & Ikebudu, 2013). Other changes in work performance include increased time to complete job tasks, particularly complex ones, problem solving, task organization or completion, time management, fatigue, and agitation (Evans, 2019; Issakainen et al., 2023; Nygård et al., 2023; Ohman et al., 2001).

An inability to maintain job demands threatens employment status, and often leads to job loss, due to low awareness and understanding of dementia in the working age population (Omote et al., 2023). Job loss often occurs before a diagnosis of young onset dementia or relatively soon after (Evans, 2019; Sakata & Okumura, 2017). Loss of employment may lead to loss of purpose or identity, disconnection from social networks made and maintained at work, plus financial uncertainty and instability (Harris & Keady, 2009). Adverse outcomes associated with job loss include higher mortality and divorce rates, alcoholism, suicide, and health problems (Stiglitz, 2002; Waddell & Burton, 2006). Conversely, maintaining employment is associated with enhanced self-esteem, health improvement, and decreased stress (Waddell & Burton, 2006). Given that people developing young onset dementia are of working age, exploring personalized, knowledge-based strategies to maintain them in employment related to their specific profile of cognitive alterations, is essential (Gure et al., 2010; Kandiah et al., 2016; Peoples et al., 2024).

Article 27 of the United Nations Convention on the Rights of Persons with Disabilities (UNCRPD) requires that persons with disabilities, including cognitive disabilities, have “the right to the opportunity to gain a living by work freely chosen or accepted in a labour market and work environment that is open, inclusive and accessible”. There are different ways an employer can approach providing workplace supports to individuals with disabilities. Whilst the UNCRPD is ratified by 177 countries worldwide, each country has its own legislative guidelines and legal frameworks. In Nordic countries Finland and Sweden, labor laws require employers to exhaust all options to retain employees before terminating their employment (Karjalainen et al., 2022). In Canada, employees hold the right to non-discrimination and fair accommodations under the human rights framework (Heron & Smith, 2020). In Canada, an essential component of employee rights is the right to non-disclosure, safeguarding individuals from having their confidential information shared without their consent (Heron & Smith, 2020). However, legislative guidelines only exist around the actual termination of employment and do not outline specific requirements to be met before employment can be terminated by an organization. As a result, it is much easier for employers to terminate employment in Canada than some other countries.

Ensuring that accommodations in the workplace align with their UNCRPD rights, could assist individuals with young onset dementia or mild cognitive impairment to sustain meaningful employment (Karjalainen et al., 2022). However, there is low awareness of dementia in the workplace (Egdell et al., 2021) and limited understanding of how to manage employees with dementia (for a review see, Peoples et al., 2024). One consequence of lack of workplace awareness is that individuals resort to developing their own strategies to mitigate their challenges (Ohman et al., 2001). These include using memos, diaries, increased time planning and organizing work tasks to facilitate their completion (Chaplin & Davidson, 2016; Ohman et al., 2001; Ritchie et al., 2018). Such strategies may be attempts to mask cognitive challenges or cope with increased pressure at work and are often short-term solutions which may increase pressure on the individual (Karjalainen et al., 2022; Peoples et al., 2024). Alternately, personalized, collaborative, and fluid solutions that meet the needs of both the individual living with cognitive challenges and the workplace are needed (Peoples et al., 2024).

**Figure 1.**
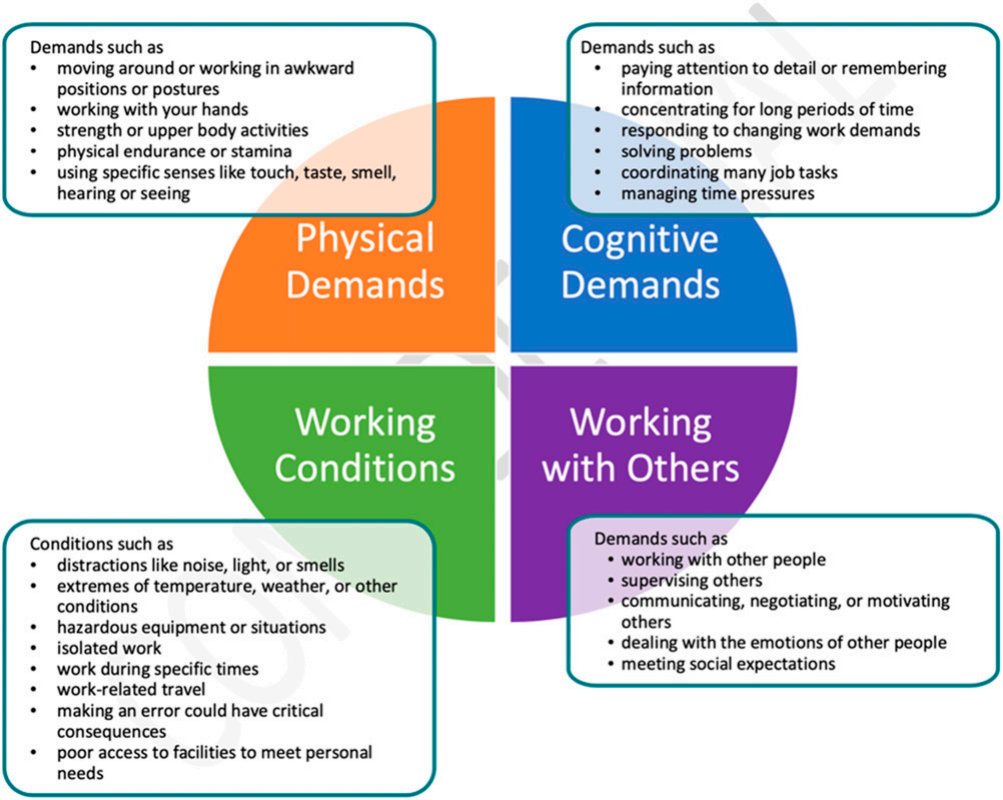
The JDAPT Framework Obtained From ACED Job Demands Tool (Gignac, Personal Communication, 2021)

Given the current lack of guidance and resources for employers and employees with young onset dementia or mild cognitive impairment to come up with individualized and effective strategies, resources developed for supporting other populations could be helpful. The Job Demands and Accommodations Planning Tool (JDAPT; Gignac et al., 2023), is an online resource developed in Canada for individuals with episodic health conditions (e.g., HIV, cancer, diabetes). The JDAPT looks at the demands of jobs in relations to four domains: physical, cognitive, interpersonal, and working conditions (Figure 1). Individuals answer questions about the different job demands they have at work, identify challenges they face in their specific jobs, and outline personalized support needs and accommodations. The JDAPT provides a structured framework to evaluate difficulties and identify areas requiring accommodations or adjustments for optimal work performance (Gignac, 2021; Gignac et al., 2023). The JDAPT aims to assist individuals with chronic health conditions to utilise more targeted accommodations and support (Gignac et al., 2023). There are three versions of the JDAPT: one for employees to self-complete, one for organizations to complete in relation to an individual employee, and a third for organizations to complete as an assessment of job role demands (Gignac et al., 2024). Within organizations the JDAPT is typically employed by HR professionals, occupational health specialists, and workplace accommodation coordinators to facilitate inclusive and sustainable employment (Gignac et al., 2023).

The JDAPT has shown early success in helping to identify specific difficulties with job demands and to facilitate accommodations for workers with episodic disabilities (Gignac et al., 2023). The JDAPT can assist workers with episodic disabilities to decide whether to disclose their illness to their employers (Gignac et al., 2021), an issue that also applies to individuals with young onset dementia or mild cognitive impairment. A recent 9-month evaluation of the JDAPT found increased self-efficacy and work productivity and reduced absenteeism among workers with chronic and episodic disabilities (Gignac et al., 2024).

As we learn more about the unique experiences of workers who develop cognitive impairments, the potential to leverage or customize existing frameworks, such as the JDAPT, is increasingly important. This study aimed to explore the potential applicability and usefulness of the JDAPT for identify job demands experienced by people with young onset dementia or mild cognitive impairment, with a view to future application to recommend personalized supports and accommodations for their workplaces.

## Methods

The data reported here were collected as part of the MCI@Work project, a cross-disciplinary international project in Canada, Finland, and Sweden. MCI@work focuses on the experiences of individuals with young onset dementia or mild cognitive impairment in the workplace from both personal, policy, practice, legal and technological perspectives. The analysis in this paper explores employees’ personal experiences, job demands, workplace challenges and support required for accommodations as they relate to the JDAPT. These three countries were selected due to their diverse policy landscapes, social welfare systems, and approaches to workplace accommodations for individuals with cognitive impairments, providing a comprehensive basis for cross-national comparison.

### Design

A qualitative descriptive approach comprising a secondary data analysis was undertaken (Ruggiano & Perry, 2019). Secondary analysis in qualitative methods involves re-examining existing qualitative data to address new research questions or delve deeper into specific themes, often through the exploration of archived interviews (Ruggiano & Perry, 2019). This approach aims to maximize the utility of rich, previously collected data, enabling nuanced insights without the need for new data collection (Ruggiano & Perry, 2019). Qualitative descriptive approaches enhance data findings for policy makers, employers, and laypeople to apply to government, work, and home settings (Sandelowski, 2000, 2010).

Ethical approval was obtained from the University Health Network Research Ethics Board in Canada, University of Eastern Finland Committee on Research Ethics (19/2018, 20/2018) and Swedish regional ethical board (file number 2018/1313–31/5). We followed the Consolidated Criteria for Reporting Qualitative Studies (COREQ) guidelines for reporting qualitative research methods (Tong et al., 2007).

### Positionality Statement

The research team was comprised of white, female researchers from Canada, Finland and Sweden. The research team was academically trained in five different disciplines: occupational therapy, psychology, law, politics and engineering. At the time of writing this article, some researchers were caregiving for someone living with dementia, although none were caregiving for someone with young onset dementia or mild cognitive impairment.

### Participants and Recruitment

Participants in the MCI@work study were included based on self-identifying: a) either a diagnosis of young onset dementia or mild cognitive impairment, in the process of seeking a diagnosis, or experiencing cognitive issues at work; b) current or recent part/full-time employment, c) capacity for written consent, d) English, Swedish and/or Finish proficiency for participation. Exclusions were significant cognitive, hearing, and language difficulties and inability to provide informed consent. Diverse participants were purposively recruited via health centers, memory clinics, partner organizations, dementia support groups, professional networks, and public events in Canada, Finland, and Sweden. Gender and geographic variation (i.e., across the three countries) were aimed for. Eligibility was confirmed, and participants received study details and risks (i.e., Participant Information Sheet, Letter of Information). They also had the opportunity to ask questions before providing written consent and were screened and scheduled for interviews by someone from the research team.

### Data Collection

The data used in this study represent portions of a larger, multi-country research program focused on understanding the lived experiences of individuals with young onset dementia and mild cognitive impairment in employment and their experiences transitioning out. Across the three countries, semi-structured interviews were conducted from April 2018 to February 2022 either in-person, via telephone, or video conferencing as per participant preferences as some data were collected during the COVID-19 pandemic. In Ontario, open-ended questions were used to explore a) personal experiences with young onset dementia or mild cognitive impairment in the workplace, including challenges they faced; b) experiences with any workplace accommodations; and c) any tools used to assist with workplace challenges. Similar interviews were held in Finland and Sweden. Participants were asked for greater detail as necessary. Interviews were conducted by a PhD-trained research assistant or post-doctoral researcher proficient in either English, Swedish and/or Finish, who were unfamiliar with the participants. All data were digitally recorded and transcribed verbatim. The Finnish and Swedish co-authors provided English translated excerpts from our broader set of interviews related to specific segments about workplace challenges. Data was translated by a bilingual professional and reviewed for accuracy by bilingual research team members. (Table 1)

**Table 1.** List of Sample Interview Questions About Workplace

Sample questions
1. Please describe your job role and workplace before and after experiencing cognitive decline 2. Please describe any physical or cognitive demands that are an important part of your job (e.g., lifting, physical endurance/stamina, recalling information, concentration, problem-solving)? 3. Please describe any conditions of your work environment that are an important part of your job (e.g., isolated work, working during specific times)? 4. Please tell me about any work adjustments that were made at work following the diagnosis 5. Please describe any learning approaches and problem-solving strategies that you used to adapt to and manage cognitive impairment in the work environment 6. Please describe any technology and/or tools that you used to support your work

In the Swedish data collection, pre-set questions were not used. Instead, key topics from each of the six questions were integrated into conversational prompts. This approach ensured that relevant themes were covered while allowing for a more flexible, participant-led discussion.

### Data Analysis

A secondary analysis using a content analysis and framework approach was undertaken to explore whether the participant experiences of workplace challenges related to the four domains of the JDAPT (Pandey, 2019; Ritchie & Spencer, 1994). Examining accommodation recommendations from the JDAPT for specific workplace challenges was beyond the scope of this analysis.

NVivo version 12.0 qualitative data analysis software (QSR International, 2018) was used to facilitate the coding process. Initially, deductive coding was informed by content analysis (Pandey, 2019), guided by predefined categories based on the JDAPT’s components, encompassing both job demands and supports (Gignac, 2021 ; Gignac et al., 2023). This process involved two PhD-trained researchers systematically sorting and categorizing participant accounts, through coding, according to the 4 domains of the JDAPT: physical and cognitive job demands, interpersonal interactions with colleagues and supervisors, and environmental factors.

Subsequently, the framework analysis method was employed to examine these coded data segments in detail, facilitating the identification of recurring patterns, contradictions, and nuanced insights within the context of the JDAPT. Beyond assessing alignment with JDAPT, we also actively sought instances where participant experiences diverged from or extended beyond the model. These discrepancies were explored in co-author discussions to assess their significance and potential implications. Three team members (BK, KMK, and AA) reviewed the coded data in team meetings, ensuring that alternative perspectives were considered. Cases where the data did not fit within JDAPT’s predefined categories were documented and analyzed to determine whether modifications to the analytic framework were necessary to capture cross-national variations or novel insights. The coded data were then systematically organized, allowing for the aggregation of similar patterns and the identification of relationships and discrepancies among different codes based on the JDAPT. The analysis was conducted sequentially, starting with data from Canada, followed by data from Finland and Sweden. This approach allowed for iterative adjustments based on emerging insights from each dataset. Instances where data did not align with the initial model (i.e., JDAPT) were carefully examined through co-author discussions to ensure consistency and accuracy. Disagreements or data that initially fell outside the model were documented and, when necessary, led to adaptations of the analytic framework to better capture cross-national nuances. This process facilitated a rigorous, contextually sensitive approach to the comparative analysis.

## Results

Twenty-three participants (11 from Canada, 7 from Finland and 5 from Sweden) participated in interviews. Participant ranges in age from 46 – 71 years of age at the time of interview (see Table 2). Multiple workplace challenges reported by the participants fell within the four JDAPT domains — *physical and cognitive job demands, interpersonal interactions, and environmental factors*. Additional information about how these were responded to by participants and their employers was also collected and is presented below in the section on Resources/Accommodations/Self-Adapted or Initiated Strategies. Quotations are delineated by the country of the participant (CAN for Canada, FIN for Finland, SWE for Sweden) and ID number.

**Table 2.** Participant Demographic Information

Participant ID	Age	Gender	Diagnosis	Employment status	Occupation
CAN P1	54	Male	MCI	Employed (seasonal)	Seasonal worker
CAN P2	57	Female	MCI	Employed (sick leave)	Training coordinator
CAN P3	62	Female	YOD	Employed (full-time)	Customer service coordinator
CAN P4	46	Male	YOD	Unemployed	Manger in economic development
CAN P5	65	Male	YOD	Retired	Support missionary
CAN P6	59	Female	YOD	Retired	Healthcare manager
CAN P7	51	Female	YOD	Employed (modified work duties)	Engineer
CAN P8	61	Male	YOD	Unemployed	Healthcare manager
CAN P9	60	Male	YOD	Retired	Electronic diagnostics/repairs
CAN P10^a^	—	—	—	—	—
CAN P11	59	Male	YOD	Unemployed	Construction
FIN P4	Over 60	Male	MCI	Employed (full-time)	Designing
FIN P5	Below 60	Female	YOD	Sick leave	Service sector
FIN P6	Below 60	Female	YOD	Retired	Care sector tasks
FIN P7	Below 60	Female	MCI	Sick leave	Financial services
FIN P8	Below 60	Female	MCI	Employed (part-time)	Developmental tasks
FIN P9	Over 60	Female	MCI	Employed (full-time)	Service sector tasks
FIN P11	Below 50	Female	YOD	Employed (full-time)	Service sector tasks
SWE P1	55	M	YOD AD	Unemployed	Service sector
SWE P2	60	F	YOD	Unemployed	Engineer
SWE P3	53	F	YOD AD	Employed but not working	Service coordinator
SWE P4	58	F	AD (Prodromal)	Employed (part time)	Administrator
SWE P5	71	M	AD	Employed (part time)	Consultant

^a^Unreported data. CAN: Canada. FIN: Finland. SWE: Sweden.

MCI: Mild Cognitive Impairment. YOD: Young Onset Dementa. AD: Alzheimers Disease.

### Domain: Physical Job Demands

**Figure 2.**
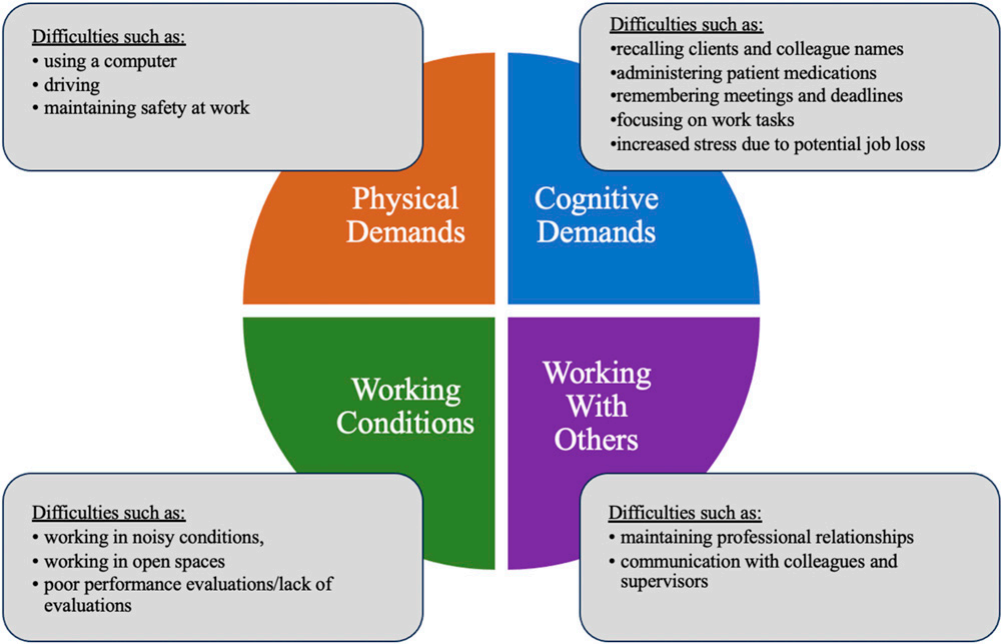
Examples of Workplace Difficulties Reported by People With YOD or MCI for Each of the Four Domains of the JDAPT

Participants described difficulties in completing their jobs when using different equipment, such as the computer (see Figure 2). For example,

“And then I became ill, and so umm I couldn’t do much, I couldn’t use the computer because umm … I could only be on it for a short period of time…I would exhaust, I would be on maybe five minutes and I would just “poof” (hands bang on table) and my head would be on my desk, it got so severe. And umm so umm any … anything with the computer was then a struggle.” (CAN Participant 10).

Fatigue interacting with cognitive challenges is another common challenge in workplaces, as people find it more effortful to complete their job tasks. For example, one participant said:

“With the diagnosis it is that ehh, yes I get tired, yes, it is above all concentration and focus which of course makes me get tired.” (SWE Participant 1).

Several participants reported difficulties related to driving, including this participant who worked in physically demanding occupation:

“a little disorientation driving, more emotional. Just subtle things. My daughter also acknowledged them to the point where, well it could maybe start to affect safety. Thirty-five feet up on a clay roof working away might not be as good a place… huge power tools and not sure how I’d react to every situation. Then I started to get some fatigue. ” (CAN Participant 5)

Another Canadian participant described specific physical challenges and the dilemma these presented for him:

“I have physical issues with the arthritis in my hand, with the back and hip problem, um, you know, with the stroke. And then from the stroke I’ve had frozen shoulders which I’m still having problems with so um, you know, with any of the heavy lifting or you know, you gotta do some manual work. I gotta watch what I do, you know, right. And I can’t say no to the work that they give me unless I turn around and I go up front with a doctor’s note and ask to be put on modified duties. But then I’ll be on permanent modified and they’re not gonna hire… No employer wants a permanent modified…when you’re in a physical job” (CAN Participants 1).

### Domain: Cognitive Job Demands

Participants reported various cognitive challenges with memory, concentration, and word-finding whilst at work. Examples encompassed challenges such as recalling messages and names from clients and colleagues, financial information, meeting details, passwords, data analytics, and various work-related tasks (see Figure 2). For example:

“I can still work but when all names of people are completely in fog. The kinds of names that I have dealt with dozens of times, they always become forgotten again. Then you seek an e-mail by using the name of the project, who is sending this. So work, effectivity at work has decreased to some extent because of it.” (FIN Participant 4)

Another reported:

“One of the things I was also responsible for was medication. As it came from the pharmacy, checking all that out, and then administering and um, I gave one of the clients the wrong medication. And uh, that was devastating for me.” (CAN Participant 6)

Additionally, difficulties might arise in navigation while driving, maintaining focus on tasks, especially in meetings involving multiple participants, and struggling to remember and articulate specific words needed for reports:

“I could not think. Like to get to the cognitive… Like, I couldn’t process, I could hardly be on the computer, I couldn’t do (pause) normal remembering things. I couldn’t calculate. I, like, I just, I, I… It’s so hard to describe. I couldn’t do those normal kinds of functions any… Like I couldn’t focus.” (CAN Participant 2).

Since symptoms of cognitive impairment are often first noticed at work and the kind of cognitive challenges faced are unexpected, these lead to worry and uncertainty. Nearly every participant mentioned taking a sick leave to investigate the root cause of their difficulties and alleviate the stress stemming from uncertainty about its long-term impact. For example:

“Dealing with the sick leave that I needed a year ago has not been very pleasant at all, and this was exacerbated at the time by the fact that I was already stressed, forgetting things due to the stress, worried about losing my job, learning about and dealing with my disability and not knowing what would happen next!” (CAN Participant 7).

Stress about what was happening to them often intensified their challenges at work. Most participants had been in the same career for several years including those which required specialized education, for example engineering. Many participants expressed major concern regarding their diminished ability to perform their job despite many years of job experience and training. CAN Participant 1 was even considering going back to school to change careers to better suit his health situation since he was only 54 at the time of the interview and potentially had 11-13 more years of work before retirement. However, the uncertainty regarding managing school due to cognitive challenges, while also juggling work responsibilities and caring for his young children, sparked significant concern and worry about the future for this participant.

“I’m trying to go back to university in [MONTH], um (pause) cause I… A couple years ago, 2013, I graduated and got a Bachelors, now trying to take it a step further. But I have all this paperwork, and it feels… I guess here’s… I feel overwhelmed all the time Like this week my wife has been working night shift… and when I get up in the morning it’s just, like it just takes so much effort to get out of bed...” (CAN Participant 1).

### Domain: Working with Others

Participants described that they often encounter(ed) interpersonal challenges at work, such as difficulties in communicating effectively with colleagues or supervisors, navigating complex social interactions, and responding appropriately to emotional or stressful situations (see Figure 2). For example,

“Felt I got more and more stressed. I had anxiety, so it made it worse, and then I would, and when I have anxiety, I avoid talking to the supervisor, where what I really needed to do was to talk to them more, so I would avoid them.” (CAN Participant 7).

Participants also reported anxiety about interactions at work. For example,

“when someone comes into my room and I'm looking at him and I always wonder what he's going to ask, what he's going to start with, and if he starts with something like that, just saying that hey, that's what we were talking about yesterday, this, that, and this, I almost have to say what we were talking about, what you mean.” (FIN Participant 4).

Other participants described difficulty with managing team dynamics, understanding non-verbal cues, or maintaining professional relations

“For me, it’s more me now asking my colleagues things. And there are times where I… I know one girl in particular, I could tell that she was sorta just getting annoyed, so I don’t ask her. And, um, we have a… There are a couple people on our floor that, um, they call them subject matter experts and, um, so that’s where I… Well one in particular, I just go to her because she’s, she understands and she’s sympathetic. She’s known me for a long time before any of this happened and so. That’s where I’ll go to get clarification and anything I need.” (CAN Participant 3).

CAN Participant 2 found that she was not supported by her employer or colleagues, which she attributed to the type of organization she worked for. She felt that private industries were further along in understanding how to help individuals with disabilities. Other participants stated that stress related to their job demands added to interpersonal challenges which often delayed them receiving accommodations, if any.

However, some participants found that their interprofessional relationships grew stronger.

“The loyalty of the other staff was just heartwarming. I can remember going to staff members saying ‘OK. I know you have your regular workload, but I need this done too’ and I'd always be fair. I'd always say, ‘well how much time do you need to accomplish this, but I need it done’ and they say, ‘well I could have it done by Friday’. And I said ‘great, but be a pal, come and see me on Friday and remind me to bring me up to speed where you're at with this and what you got done’. And on Friday, just like clockwork, they walked into my office and said you asked me to remind you about this.” (CAN Participant 8).

A participant from Sweden reported a similar arrangement. .

“I had an agreement with them [co-workers]; you must tell my boss if you think I don’t physically function good enough, and this worked out well for a couple of years --- I told them that they have to be able to use me in the best way and trust that I do things right.” (SE Participant 2).

### Domain: Working Conditions

Participants also struggled to meet project deadlines and work in noisy conditions, such as a large open space with multiple cubicles (see Figure 2). For example:

“I am overwhelmed very quickly, but don’t put me in a cubicle, like so many people are sitting in cubicles because that will put me over the edge.” (CAN Participant 10).

CAN Participant 7 noticed challenges working in an office space after they had the experience working from home during the COVID-19 pandemic where:

“I don't have people running in front of me, or talk, having a discussion right beside me. I don't have the fear of my supervisor in the next cubicle over wondering what he's thinking about me [laughter].”

Difficulties faced completing job demands were also due to an inability to drive to work, due to impaired executive functioning, lack of resources, and/or poor comprehension. For example,

“Suddenly I got a call back that says no, just take, take that time off ‘cause I wasn’t given the approval to go ahead, uh, to, to, to work or drive in you know.” (CAN Participant 1).

As part of most company policies, employers are responsible for providing performance evaluations for their employees. Participants with cognitive impairment described negative experiences with feedback meetings and performance reports regarding their capabilities and accommodations, if they were provided at all. For example, one participant stated:

“[Program office rep] also suggested that we meet with my supervisor [Name] to talk about the problems with me understanding what he wanted, and that sort of thing, and he never, he never agreed to that. He didn't say anything, he didn't ever respond to her. So, I thought that was kind of sad because things could have improved a little faster.” (CAN Participant 7).

Some participants stated that their performance meetings and evaluations were not properly discussed with them and were mostly documented to either provide grounds for removal of accommodations, prove that the participant could not perform their job and should be terminated, or to try and prevent the participant from coming back to work.

“Well pretty much I was put on sick leave. And then out [of work] after that.” (FIN Participant 5)

### Resources/Accommodations/Self-Adapted or Initiated Strategies

In addition to identifying job demands within the four domains, the JDAPT can also provide resources and suggest accommodations for the individual. Whilst this was not within scope of the current analysis, the participants reported using self-adapted strategies in the absence of ones provided by their employer. These strategies included a variety of tools to remember information including pen and paper notes, including sticky notes, notebooks, emails, and setting timers to complete tasks. Some relied more heavily on their work and personal calendars to help them remember important information, or would ask colleagues to remind them of meetings, deadlines, and other work tasks. While most participants preferred using the traditional method of writing down notes/reminders on paper or being given a list of things to do from their employer; others used electronic tools (e.g. alarms, desktop computer, e-mail, smartphone) and other strategies such as a daybook, or color coding their e-calendars.

“I found that I had to rely more and more on all kinds of notepads. I carried a kind of notebook with me all the time, I wrote all the things in it all the time.” (FIN Participant 4).

Some individuals developed their own organizational system, using colour coding or placing more important information in a highly visible area of their desk. For example, CAN Participant 7 would write and send a follow-up email to her boss after their meetings to summarize the information discussed, so she could access this information later. CAN Participant 2 mentioned verbally rehearsing information until she could write it down:

“It’s almost like it’s gone from nice to do and helpful to do, this is really gonna help you cognitively, to now it’s like survival sometimes, right? But let’s say…I’m looking up the price of something, so it’s four dollars and nineteen cents, and I have to go from the family room to my office…, and input that amount of money because that’s what I just spent on something, okay? I gotta put it in the budget. So, when I leave, I start saying four nineteen, four nineteen, four nineteen, four nineteen, four nineteen. I sit down, four nineteen, four nineteen, and I just keep repeating it….And then once I get there and/or if there’s paper around, then I’ll grab the piece of paper and I’ll rip it and it’ll come with me.”

Another participant reported using photographs in their phone to help remember people, by linking a face to a name:

“Then I wrote up her name on the phone number so that I know what to look up and then it is so that I take pictures, I will take a picture of you also with your phone number saying that, so that I know that before, it is something that can help...” (SWE Participant 2).

Participants also mentioned several strategies to manage the interpersonal challenges they were experiencing. FIN Participant 4 asked their supervisor to allow them to:

“move on to easier tasks and away from this managerial position, that I’d like to return to my original profession as a line designer.”

Some participants had their work duties were modified, such as reducing workload, modifying deadlines, or a change in job description. CAN Participant 7 who worked for the government had access to a union that was able to help guide her through interacting with her employer, knowing her rights, and receiving the accommodations she needed.

“I was given more time to work, I was given a few like, I wasn’t given real deadlines like I’m not, a lot of the work it has to be done that day, and the work that I’m doing doesn’t. So, they accommodated me in that way.”

The type of job and workplace conditions appeared to influence whether participant’s employers provided adequate accommodations to address their workplace challenges, or whether participants were left on their own to figure out appropriate supports.

“I find it very important to stress here that they [employer] were incredibly good to me. They could have said “I’m sorry but we have nothing that you are capable of doing here.”…but they did not. But I’m incredibly grateful that I got that possibility, actually. And I also understand that they have looked outside the boxes to make it work… eh… and I, actually, I’m very impressed that they did this.” (SWE Participant 4).

## Discussion

This study explored the applicability of the Job Demands and Accommodations Planning Tool (JDAPT: Gignac et al., 2021) to the workplace challenges faced by people with young onset dementia and mild cognitive impairment. The framework analysis revealed that individuals living with cognitive decline due to young onset dementia or mild cognitive impairment experience similar job demands and challenges to individuals working with episodic health conditions. As such their experiences could be mapped onto the four domains of the JDAPT (see Figure 2): physical, cognitive, interpersonal, and working conditions, confirming it as a feasible tool for individuals with young onset dementia or mild cognitive impairment in the workforce.

Taking each domain in term, participants noted changes to their stamina and physical capacity. This affected their ability to perform job related tasks such as long stretches on a computer, driving to their workplace or as part of their job. Concern about physical capacity also created safety concerns on the job. The JDAPT physical demands section also focuses on aspects such as heavy lifting, working with your hands, changes to your senses, and physical endurance or stamina. Participants described fewer physical challenges at work compared to the other three domains of the JDAPT. This may be because most of the participants did not have physically demanding jobs but also due to young onset dementia and mild cognitive impairment being primarily cognitive rather than physical disorders. Participants described challenges completing job tasks due to memory decline, difficulties focusing, and increased stress. Remembering information, concentration, and managing time pressures (e.g., stress) are all aspects of the JDAPT that are applicable for individuals with young onset dementia or mild cognitive impairment.

Many participants also expressed difficulties managing professional relationships and communication with colleagues and supervisors. This often stemmed from anxiety and stress from having to discuss challenges they were experiencing with co-workers. These experiences align with both the communication and working with others aspects of the JDAPT. Participants with young onset dementia and mild cognitive impairment also reported difficulties working in noisy, open spaces with distractions, which aligns with the environmental domain of the JDAPT. Performance evaluations or ineffective evaluations were another concern raised by participants. Although performance evaluations are not directly noted in the JDAPT, poor access to facilities to meet personal needs is listed under Working Conditions. Therefore, performance evaluations can serve to check-in with employees and make any recommendations for accommodations to meet personal needs as found in the JDAPT.

In addition to confirming the potential of the JDAPT for those with cognitive impairments in the workforce, the interviews revealed how these employees currently manage their workplace challenges. Essentially, they rely on self-adapted strategies and accommodations to meet workplace difficulties in the four domains of the JDAPT. The current findings highlight innovative solutions derived from existing strategies to address workplace challenges that could be incorporated into the JDAPT recommendations. For example, participants reported utilizing memory aids such as calendars, emails, and notebooks, and adjusting work schedules and tasks to meet their specific needs. The theme of self-adapted strategies is not novel but provides further evidence on the types of strategies and accommodations people with young onset dementia and mild cognitive impairment make at work (Chaplin & Davidson, 2016; Ohman et al., 2001; Ritchie et al., 2018; Karjalainen et al., 2022, Nygård et al., 2021). The JDAPT’s systemic approach could suggest new strategies and accommodations that may better suit individual’s specific needs.

Participants in the current study often used these self-adapted strategies because of a lack of support and guidance from their employer on how to best manage their cognitive challenges at work. The findings add further insights into how the workplace setting has a large impact on whether support and accommodations are made available by the employer (Chaplin & Davidson, 2016; Ohman et al., 2001; Ritchie et al., 2018). Thus, the out of the JDAPT could help to guide conversations with employers on accommodations. Fostering continuous communication between the employee and employer regarding accommodations is crucial (Gignac et al., 2023), especially since cognitive changes may fluctuate over time (Peoples et al., 2024). Information about the JDAPT could be included in guidance about dementia friendly workplaces (e.g. UK Alzheimer’s Society, 2015). This can help to address the lack of awareness by employers and employees in understanding how to manage cognitive challenges in the workplace (Egdell et al., 2018; Peoples et al., 2024).

The inclusion of multiple national contexts in this research provided important insights into the practical and policy differences in supporting individuals with dementia in employment. Variations in workplace accommodation policies, legal protections, and cultural attitudes toward disclosure and support shaped participants’ experiences across countries. For instance, in Sweden, there is a strong emphasis on state-supported accommodations, where employers are legally obligated to provide workplace modifications, which contrasts with the more self-advocacy-driven approaches seen in Canada. Finland, on the other hand, features a mix of employer responsibility and public sector support, reflecting its unique social welfare system. These differences in policy and practice highlight the influence of national frameworks on workplace support for people with dementia, underscoring the need for context-sensitive approaches in employment and accommodation strategies across different countries.

Taking the findings together, the JDAPT has strong potential to support individuals with dementia or mild cognitive impairment in maintaining employment. However, its practical implementation requires careful consideration. To ensure accessibility, information about the tool could be disseminated through healthcare providers, occupational health, and workplace disability support services. These professionals can help identify individuals who might benefit from using the JDAPT, facilitate access and support individuals in translating the results into practical workplace adjustments. Individuals could receive guidance effectively communicating the recommended accommodations to employers, ensuring that adjustments are implemented and that ongoing support is provided to maintain work participation. By integrating the JDAPT within a broader framework of vocational support, it could become a valuable tool for enhancing work sustainability for individuals with dementia or mild cognitive impairment.

### Limitations

Due to difficulties recruiting working individuals with young onset dementia or mild cognitive impairment, particularly during the global COVID19 pandemic, the sample potentially lacks diversity of employment types and may not fully capture the varied experiences and challenges individuals experience in different employment contexts. Additionally, while the study explores some demographic factors, the influence of cultural, gender, and socio-economic differences on the outcomes cannot be fully addressed. Limited employment types and consideration of socio-demographic variables limit the generalizability of the findings to other workforce segments and populations. This study intentionally sought variation in gender and geography to capture diverse workplace experiences but did not explicitly aim to examine differences based on age or ethnicity, which may limit the transferability of findings across broader demographic groups. Due to difficulty recruiting participants diagnosed with young onset dementia or mild cognitive impairment, some participants were self-identifying individuals with cognitive impairments. However, the majority were recruited from memory assessment clinics or services for people with young onset dementia or mild cognitive impairment, such as those offered by dementia charities in the three countries.

## Conclusion

The JDAPT has potential to address the workplace difficulties encountered by individuals with young onset dementia or mild cognitive impairment. Future work piloting the JDAPT framework in real-world workplace settings is required to assess its feasibility and effectiveness in supporting individuals with young onset dementia and mild cognitive impairment to maintain employment. Additionally, exploring its integration within existing workplace policies could help ensure that accommodations are systematically adopted to foster inclusive work environments.
